# Bone-targeting exosome nanoparticles activate Keap1 / Nrf2 / GPX4 signaling pathway to induce ferroptosis in osteosarcoma cells

**DOI:** 10.1186/s12951-023-02129-1

**Published:** 2023-09-30

**Authors:** Wenkai Chen, Zongguang Li, Naichun Yu, Linlin Zhang, Hongyu Li, Yongjie Chen, Fengqing Gong, Wenping Lin, Xu He, Siyuan Wang, Yue Wu, Guangrong Ji

**Affiliations:** 1 Department of Orthopedic Surgery, Xiang’an Hospital of Xiamen University, School of Medicine, Xiamen University, Xiamen, China; 2 Fujian Provincial Key Laboratory of Organ and Tissue Regeneration, Xiamen Key Laboratory of Regeneration Medicine, Organ Transplantation Institute of Xiamen University, School of Medicine, Xiamen University, Xiamen, China; 3 Department of Spine Surgery, Shenzhen Pingle Orthopedic Hospital, Affiliated Hospital of Guangzhou University of Traditional Chinese Medicine, Shenzhen, China; 4 Department of Orthopedic Surgery, Zhongshan Hospital, School of Medicine, Xiamen University, Xiamen, China; 5 Department of Pathology, Zhongshan Hospital, School of Medicine, Xiamen University, Xiamen, China

**Keywords:** Homologous targeting, Ferroptosis, Osteosarcoma, Chemotherapy, Nrf2

## Abstract

**Background:**

In recent years, the development of BMSCs-derived exosomes (EXO) for the treatment of osteosarcoma (OS) is a safe and promising modality for OS treatment, which can effectively deliver drugs to tumor cells in vivo. However, the differences in the drugs carried, and the binding of EXOs to other organs limit their therapeutic efficacy. Therefore, improving the OS-targeting ability of BMSCs EXOs and developing new drugs is crucial for the clinical application of targeted therapy for OS.

**Results:**

In this study, we constructed a potential therapeutic nano platform by modifying BMSCs EXOs using the bone-targeting peptide SDSSD and encapsulated capreomycin (CAP) within a shell. These constructed nanoparticles (NPs) showed the ability of homologous targeting and bone-targeting exosomes (BT-EXO) significantly promotes cellular endocytosis in vitro and tumor accumulation in vivo. Furthermore, our results revealed that the constructed NPs induced ferroptosis in OS cells by prompting excessive accumulation of reactive oxygen species (ROS), Fe^2+^ aggregation, and lipid peroxidation and further identified the potential anticancer molecular mechanism of ferroptosis as transduced by the Keap1/Nrf2/GPX4 signaling pathway. Also, these constructed NP-directed ferroptosis showed significant inhibition of tumor growth in vivo with no significant side effects.

**Conclusion:**

These results suggest that these constructed NPs have superior anticancer activity in mouse models of OS in vitro and in vivo, providing a new and promising strategy for combining ferroptosis-based chemotherapy with targeted therapy for OS.

**Supplementary Information:**

The online version contains supplementary material available at 10.1186/s12951-023-02129-1.

## Background

Osteosarcoma (OS) is an aggressive malignancy of mesenchymal cell origin that primarily affects children and young adults [1]. Bone mesenchymal cells that are cancerous in nature have the ability to proliferate rapidly and form bone tissue that resembles a tumor, ultimately leading to mortality in humans [2].To date, in addition to traditional surgery, chemotherapy, and immunotherapy, targeted drug therapy is considered one of the most effective strategies in clinical applications to highlight a cure for malignant OS [3].

BMSCs-derived exosomes (EXOs) have been developed for targeted therapy of OS because of their “homing effect” on OS cells [4]. However, the binding of BMSCs-derived EXOs to other organs in vivo has limited their targeting to OS [5]. With the rapid development of nanotechnology, nanoparticle (NP) surface functionalization using EXOs presents a potential targeting approach [6]. Modification of EXO surfaces using bone-targeting peptides has been shown to direct EXO aggregation in bone in vivo [7]. Therefore, based on this emerging approach, we surface antigenically modified MSCs-derived EXOs with bone-targeting peptides (SDSSD) to improve cellular endocytosis in vitro and tumor accumulation in vivo.

The cargo carried in EXOs is essential for killing tumors. In our studies of targeted NPs for the treatment of OS, it is surprising and exciting that bone-targeting exosomes (BT-EXO) carrying capreomycin (CAP), a traditional anti-tuberculosis drug, induced ferroptosis in OS cells. At this stage, ferroptosis is recognized as a promising strategy of cell death for effective tumor eradication due to tumor heterogeneity, tumor cell phenotypic diversity, and resistance to apoptosis induced by chemotherapeutic agents [8]. Inhibition of glutathione peroxidase 4 (GPX4) for defective lipid peroxidation repair, excessive accumulation of reactive oxygen species (ROS), and the Fenton reaction are key aspects of ferroptosis [9].

Our results indicated that our prepared NPs efficiently targeted OS in vivo, exerted significant cytotoxic effects through ferroptosis, and significantly inhibited tumor growth in vivo with no significant side effects. In addition, our delved into the potential mechanism by which CAP induces ferroptosis, likely via the activation of the Keap1/Nrf2/GPX4 pathway. This pathway serves as the foundation for the efficacy of our NPs in inhibiting OS.

## Results

### Generation and characterization of BT-EXOs-CAP

**Fig. 1:**
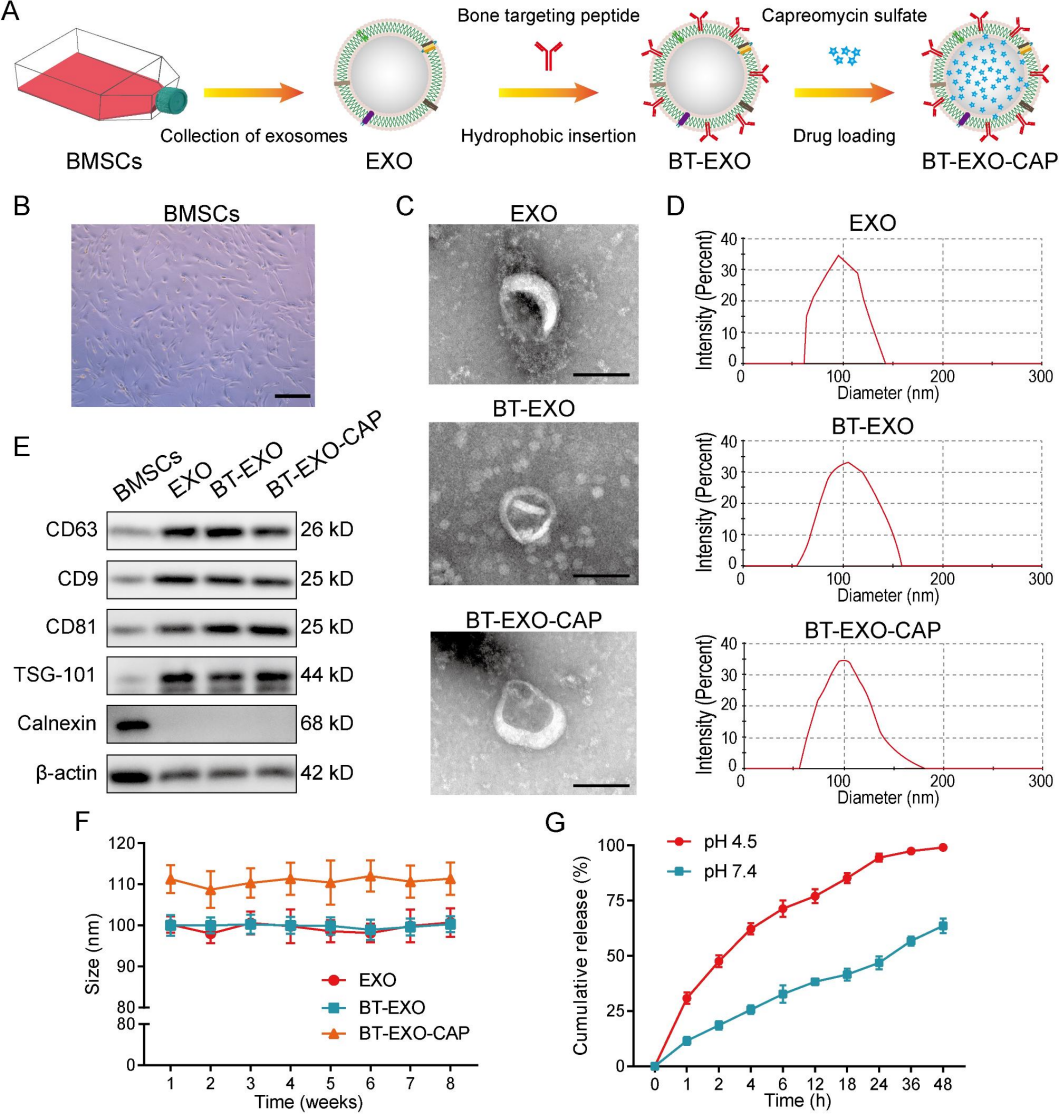
Preparation and characterization of BT-EXOs-CAP. (**A**) Preparation flow chart of bone targeting NPs. (**B**) Representative images of BMSCs. (**C**) Representative TEM images of EXOs, BT-EXOs, and BT-EXO-CAP. (**D**) Size distribution of EXO, BT-EXO, and BT-EXO-CAP. (**E**) WB analysis of EXO markers CD63/9/81, TSG101, endoplasmic reticulum marker, and Calnexin on BMSCs, EXO, BT-EXO, and BT-EXO-CAP. (**F**) Particle size by NP tracking analysis for EXO, BT-EXO, and BT-EXO-CAP during storage at -20℃. (**G**) The drug release profile of BT-EXO-CAP in PBS with pH 4.5 and 7.4. Scale bar: B 200 μm, C 100 nm

We used ultracentrifugation to isolate EXOs from the BMSCs culture medium, labeled bone targeting peptide (SDSSD) on EXOs by hydrophobic insertion, and then used ultra-speed oscillation to promote CAP into BT-EXO (Fig. 1A-B). EXO, BT-EXO, and BT-EXO-CAP were characterized by TEM and size distribution analysis (Fig. 1C-D). The WB results revealed that EXO, BT-EXO, and BT-EXO-CAP exhibited expression of EXO-specific antigens, namely TSG101 and CD9/63/81, while lacking expression of the endoplasmic reticulum-specific antigen, calnexin, and exhibiting low β-actin expression (Fig. 1E). After 8 weeks of storage at-80 °C, the average particle size of BT-EXO was maintained at 100 nm, while that of BT-EXO-CAP was maintained at 110 nm (Fig. 1F).

The present study investigated the drug release kinetics of BT-EXO-CAP at pH 7.4 and pH 4.5, considering the weakly acidic microenvironment of OS, revealing that the BT-EXO-CAP drug release rate was significantly accelerated under weakly acidic conditions (Fig. 1G).

### BT-EXOs loading promoted CAP targeting of OS

**Fig. 2:**
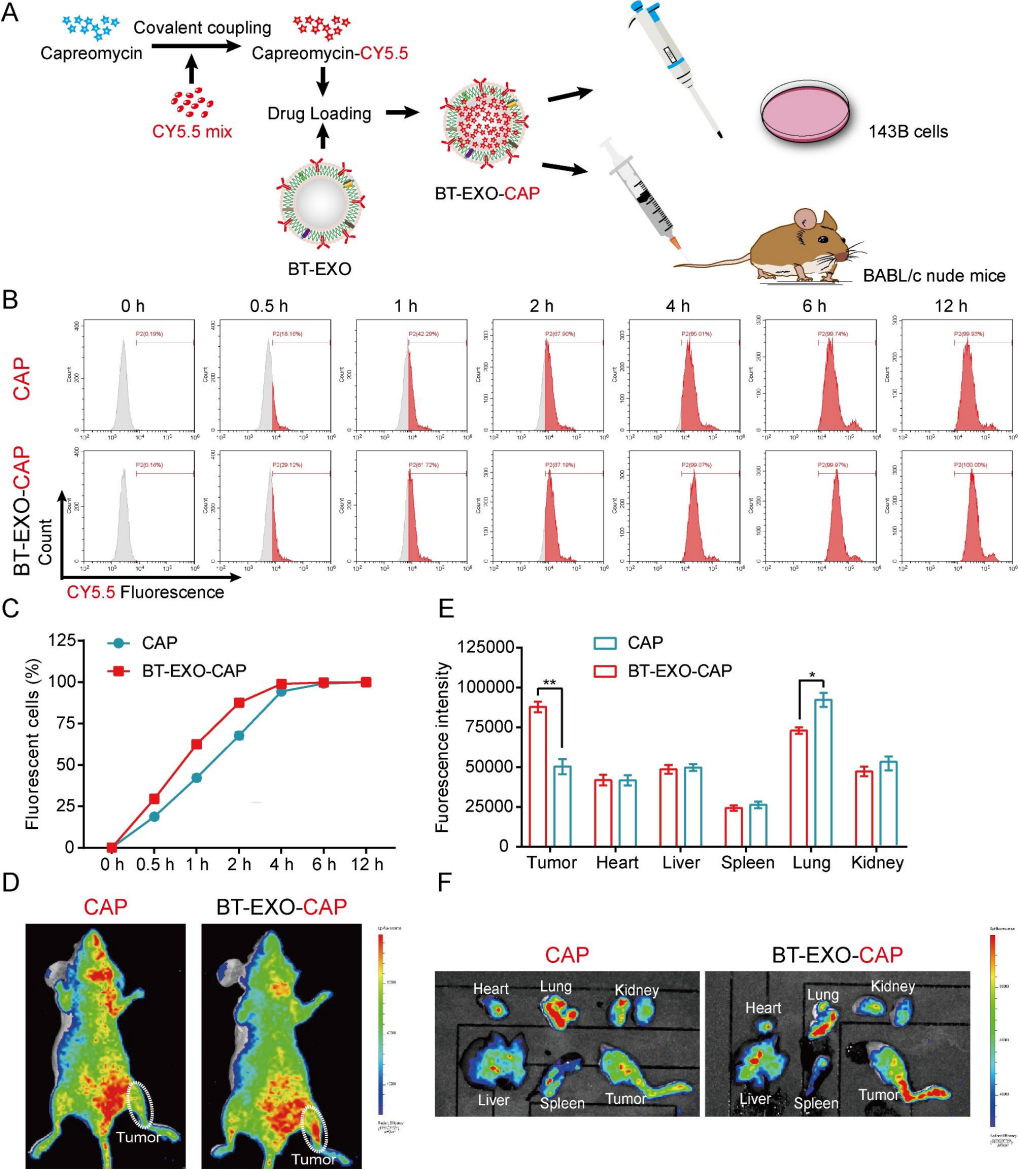
BT-EXOs loading promoted CAP targeting of OS. (**A**) The experimental schematic illustration. (**B**-**C**) BT-EXOs loading accelerated CAP entry into OS cells. Detection of cell fluorescence by flow cytometry using CY5.5. n = 3/group. (**D**) Representative fluorescence imaging of both tumor-bearing mice administrated various treatments and (**E**-**F**) mice organs. n = 3. * p < 0.05, ** p < 0.01

In order to achieve CAP drug tracing, we used CY5.5 to fluorescently label CAP and then loaded it into BT-EXO (Fig. 2A). In vitro, BT-EXO loading accelerated the entry of CAP-CY5.5 into OS cells (Fig. 2B-C). In vivo, BT-EXO loading helps CAP-CY5.5 target OS and reduces the accumulation of CAP-CY5.5 in the lungs (Fig. 2D-F). In addition, we compared the effects of EXO-CAP and BT-EXO-CAP on the survival rate of OS cells and found that BT-EXO-CAP had a more significant killing effect (Fig. S1).

### Anti-OS effect of BT-EXO-CAPin vitro

**Fig. 3:**
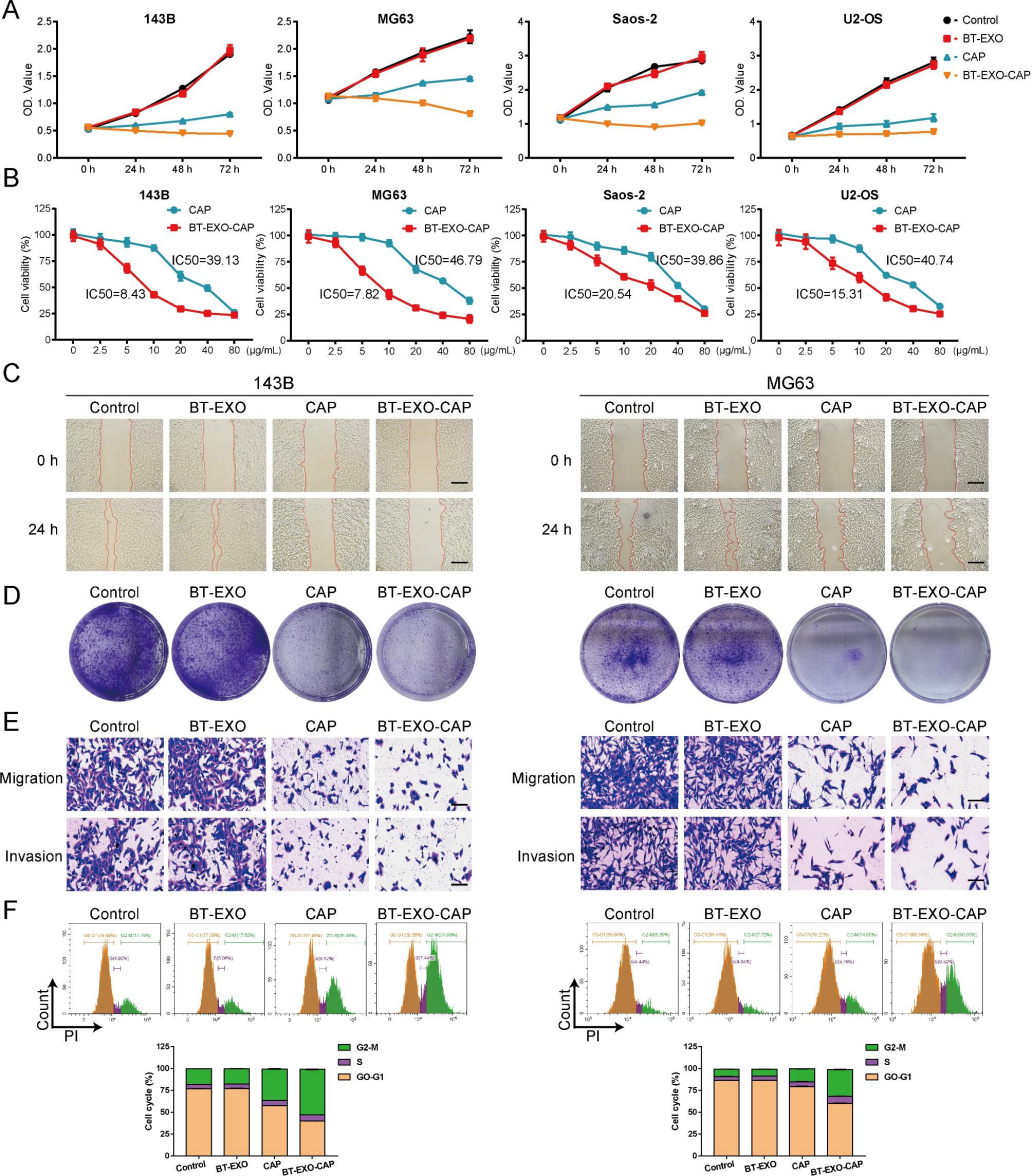
BT-EXO-CAP inhibited human OS cell proliferation, colony formation, and invasion. (**A**) Cell viability of OS cells treated with PBS, BT-EXO, CAP, and BT-EXO-CAP. (**B**) Cell viability of OS cells treated with multiple concentrations of CAP and BT-EXO-CAP. (**C**) Representative images of both cell wound scratch assay and (**D**) OS cell clonogenicity. (**E**) Representative Transwell-images of OS cell migration and invasion. (**F**) Detection of the OS cell cycle through flow cytometric. n = 3. Scale bar: C 200 μm, E 100 μm

We compared the effects of BT-EXO, CAP, and BT-EXO-CAP on the survival rate of four OS cell lines after incubation with drugs for 72 h and found that BT-EXO-CAP significantly inhibited the survival of OS cells (Fig. 3A). Meanwhile, we compared the half maximal inhibitory concentration of CAP and BT-EXO-CAP, and found that the IC50 of BT-EXO-CAP was significantly lower than that of CAP (Fig. 3B). BT-EXO-CAP inhibited proliferation, colony formation and invasion of human OS cells (Fig. 3C-E) (Fig. S2-4). Flow cytometry also found that BT-EXO-CAP induced cell cycle arrest at the G2/M stage (Fig. 3F) (Fig. S5).

**Fig. 4:**
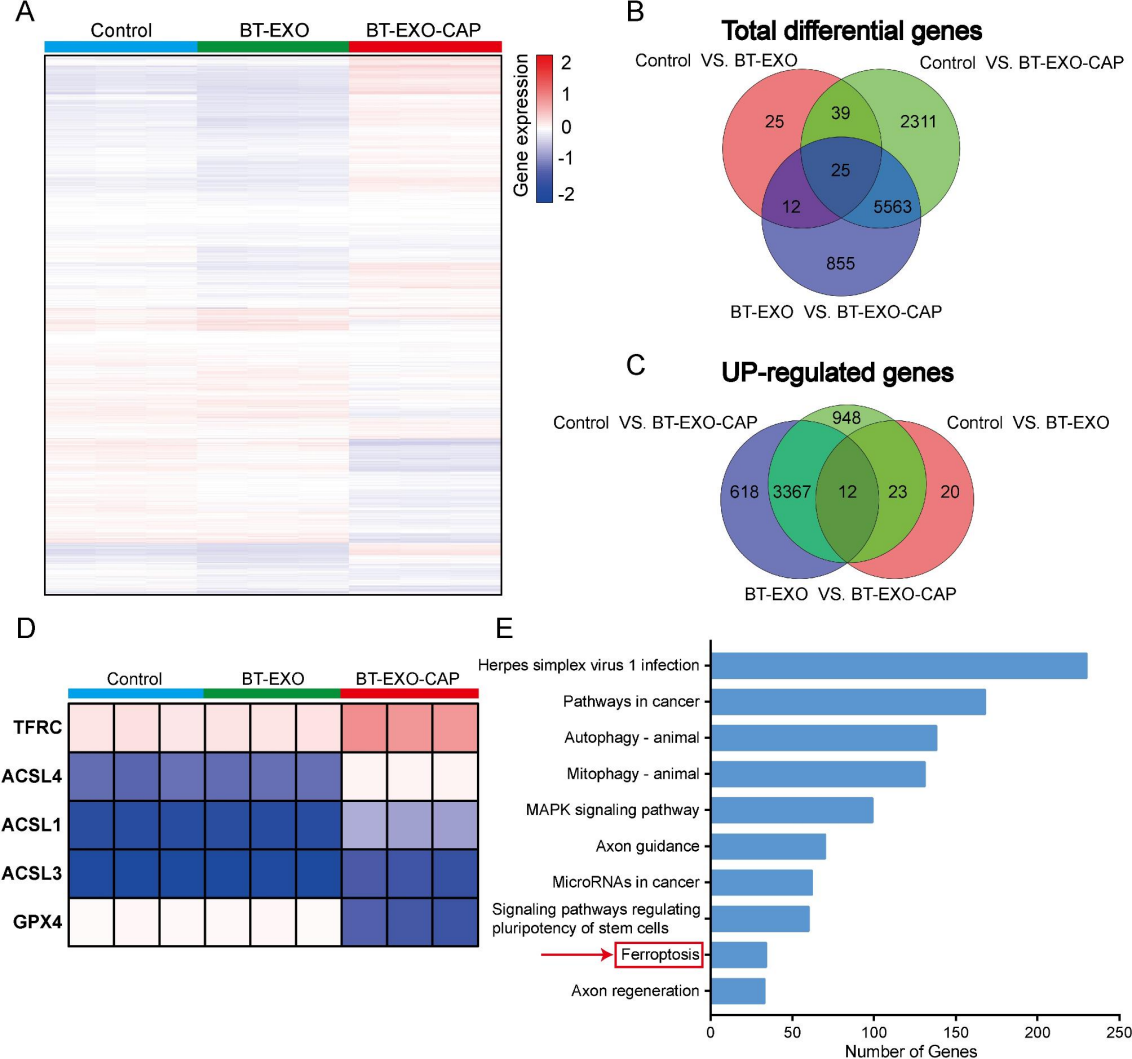
RNA-Seq showed that BT-EXO-CAP promoted ferroptosis in 143B cells. (**A**) Visualization of differentially expressed genes (DEGs) in cells treated with PBS, BT-EXO, and BT-EXO-CAP using a heatmap. n = 3. (**B**-**C**) Venn diagram of total DEGs and overexpressed genes. (**D**) RNA expression of ferroptosis-related genes. (**E**) Analysis of altered genes in top categories relying on KEGG from BT-EXO vs. BT-EXO-CAP (fold change ≥ ± 2 & P -value < 0.05)

### BT-EXO-CAP induced ferroptosis in OS cells

**Fig. 5:**
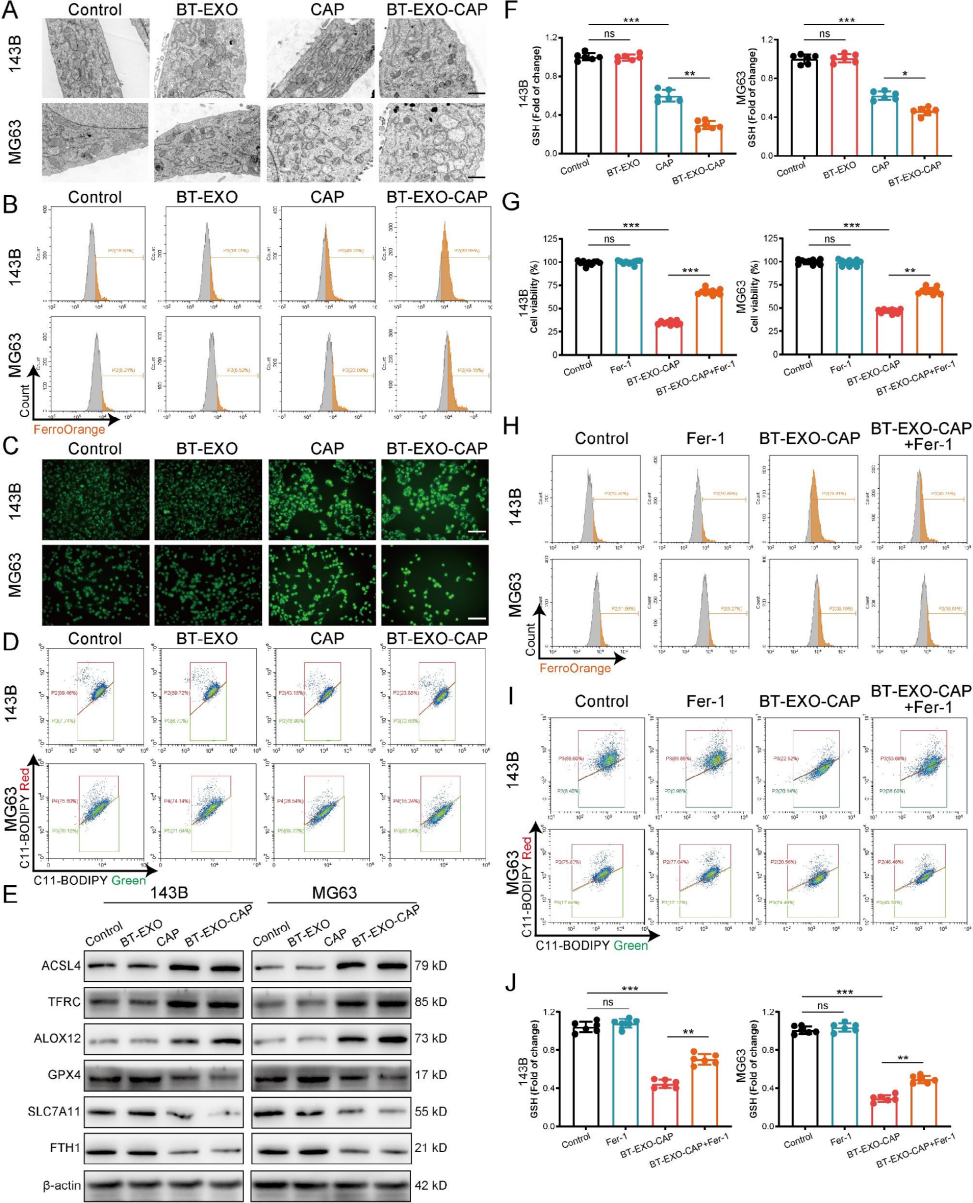
BT-EXO-CAP induced ferroptosis in human OS cells. (**A**) Representative TEM picture of mitochondria. (**B**) Flow cytometry-based detection of FerroOrange fluorescence in OS cells. (**C**) DCFH-DA fluorescence of OS cells captured by fluorescence microscope. (**D**) Flow cytometry-based detection of C11-BODIPY fluorescence in OS cells. (**E**) Measurement of ferroptosis-related protein expressions via WB. (**F**) GSH levels in OS cells. n = 6. (**G**) Cell viability of OS cells. n = 10. (**H**) Flow cytometry-based detection of FerroOrange and (**I**) C11-BODIPY fluorescence in OS cells. (**J**) GSH levels in OS cells. n = 6. * p < 0.05, ** p < 0.01, *** p < 0.001. Scale bar: A 1 μm, C 100 μm

The RNA-Seq analysis indicated the probable role of mitophagy (Fig. 4A-E), and mitochondria could be seen smaller, the double membrane density increased, the mitochondrial ridge disappeared, and the outer membrane broke after BT-EXO-CAP treatment by TEM (Fig. 5A). BT-EXO-CAP induced intracellular Fe2 + concentration increase and ROS accumulation (Fig. 5B-C), and promoted lipid peroxidation of the cell membrane (Fig. 5D) (Fig. S6). WB showed that BT-EXO-CAP overexpressed ACSL4, TFRC, and ALOX4 and suppressed GPX4, SLC7A11, and FTH1(Fig. 5E). BT-EXO-CAP decreased GSH concentration in OS cells (Fig. 5F). These results suggest that BT-EXO-CAP promotes ferroptosis in OS.

Ferroptosis agonist (Fer-1) reversed the survival inhibition of BT-EXO-CAP on OS (Fig. 5G), reduced intracellular Fe2 + concentration (Fig. 5H), prevented cell membrane lipid peroxidation (Fig. 5I) (Fig. S7) and increase GSH concentration (Fig. 5J).

### BT-EXO-CAP activated the Keap1 / Nrf2 / GPX4 signaling pathway leading to ferroptosis in OS cells

**Fig. 6:**
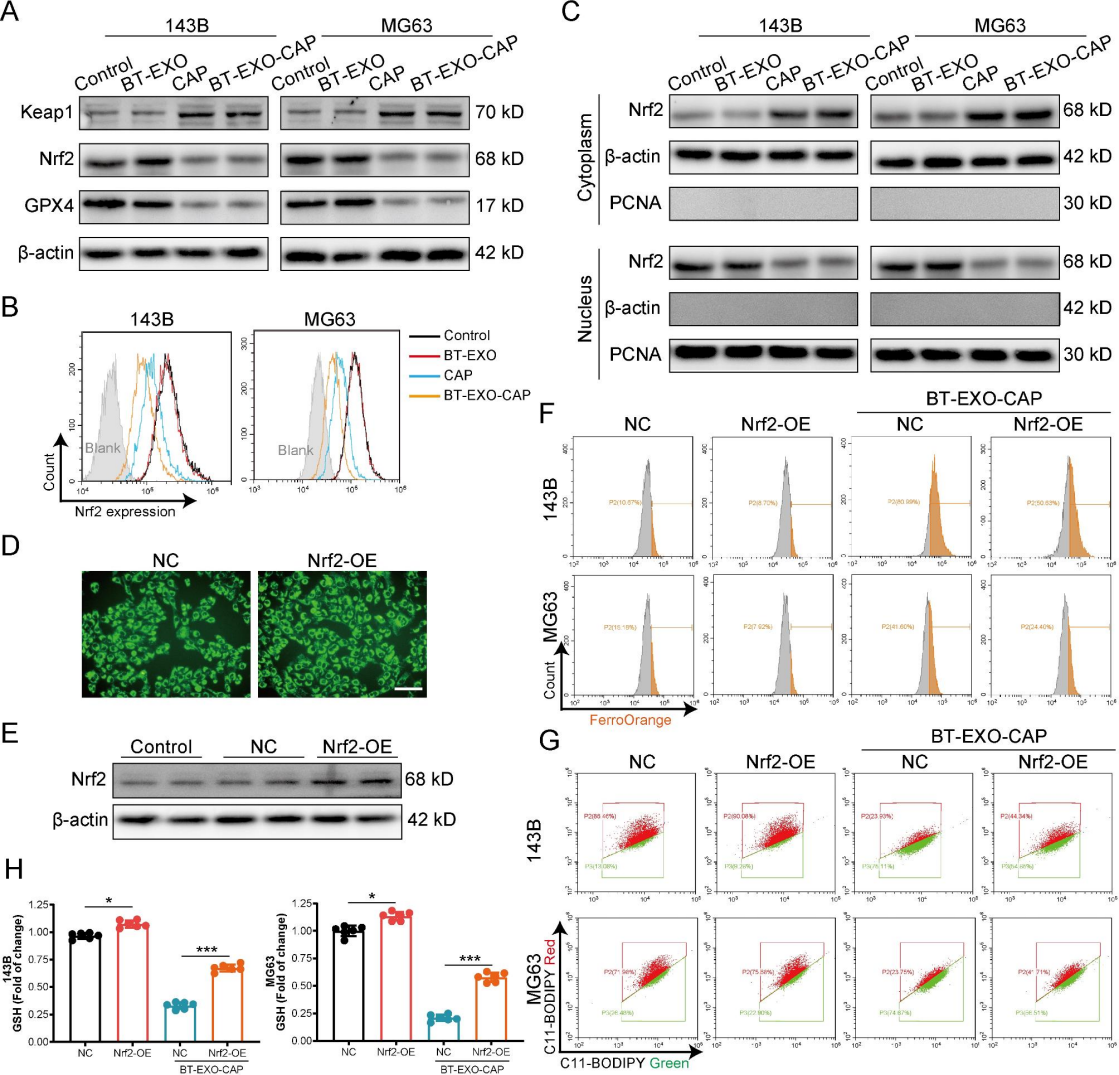
BT-EXO-CAP activated the Keap1 / Nrf2 / GPX4 signaling pathway leading to ferroptosis in OS cells. (**A**) Representative immunoblot of Keap1, Nrf2, and GPX4 protein levels. n = 3. (**B**) Flow cytometry-based detection of Nrf2-expression. n = 3. (**C**) Representative immunoblot of Nrf2 protein levels in cytoplasm and nucleus. n = 3. (**D**) The fluorescence microscope captures the FITC fluorescence of OS cells. (**E**) Immunoblot analysis of Nrf2 in OS cells. n = 3. (**F**) Flow cytometry-based detection of FerroOrange and (**G**) C11-BODIPY fluorescence in OS cells. (**H**) GSH levels in OS cells. n = 6. * p < 0.05, *** p < 0.001. Scale bar: D 100 μm

Nrf2 is an important upstream transcription factor that governs GPX4 expression [10]. BT-EXO-CAP upregulated the expression of the Nrf2-antagonist Keap1 and downregulated Nrf2 and GPX4 (Fig. 6A-B). Further, BT-EXO-CAP was found to inhibit the entry of Nrf2 from the cytoplasm into the nucleus (Fig. 6C). We constructed Nrf2 overexpressing 143B cell lines (Fig. 6D-E) using lentivirus and found that Nrf2 overexpression effectively reversed BT-EXO-CAP-induced Fe2 + aggregation (Fig. 6F), lipid peroxidation (Fig. 6G) (Fig. S8) and GSH reduction (Fig. 6H).

### The efficacy of BT-EXO-CAP against OS in vivo

**Fig. 7:**
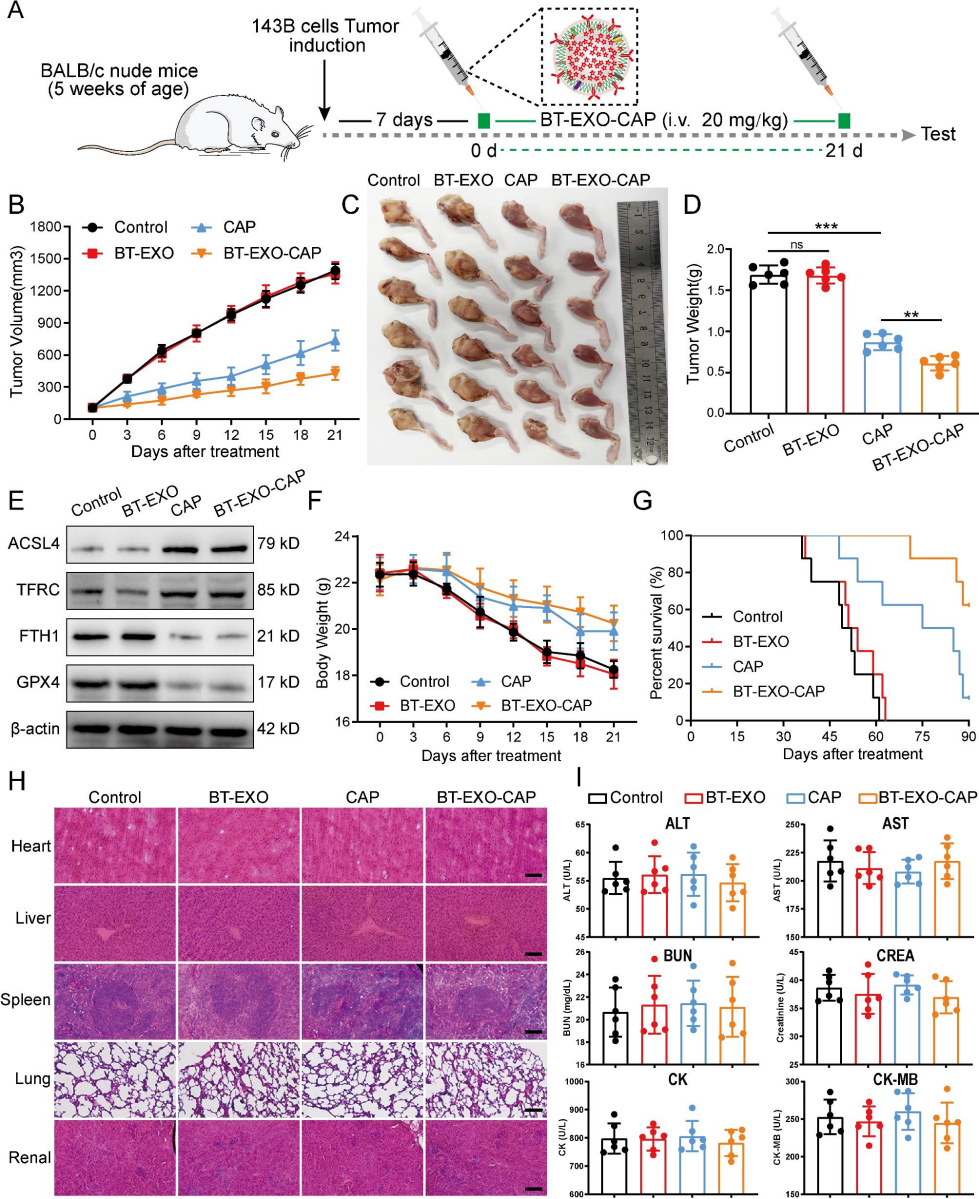
Therapeutic efficacy of BT-EXO-CAPin vivo. (**A**) The experimental schematic illustration. (**B**) Tumor growth curves of tumor volume following 21 days of various treatments. (**C**-**D**) Tumor images and weight of mice administrated various treatments following 21 days. (**E**) Immunoblot analysis of ACSL4, TFRC, FTH1, and GPC4. (**F**-**G**) Weight changes and survival rates of mice administered various treatments. (**H**) Representative pictures of HE staining of mice organs. (**I**) ELISA kit-based detection of ALT, AST, BUN, CREA, CK, and CK-MB levels. n = 6. ** p < 0.01, *** p < 0.001. Scale bar: H 100 μm

The study involved the administration of BT-EXO-CAP in a continuous manner for 21 days, commencing 7 days after the implantation of 143B tumor cells in the 5-week-old nude mice (Fig. 7A). The results revealed that the inhibitory effect of BT-EXO-CAP on OS growth was found to be significantly more potent than that of CAP (Fig. 7B-D). The BT-EXO-CAP and CAP-treated groups exhibited FTH1 and GPX4 suppression and ASCL4 and TFRC overexpression, suggesting the promotion of ferroptosis (Fig. 7E).

Mice that were administered with BT-EXO-CAP and CAP exhibited a significant weight loss compared to the controls (Fig. 7F) and demonstrated prolonged survival (the mice euthanized upon reaching a tumor volume of 2000 mm^3^). The BT-EXO-CAP group exhibited the highest survival rates (Fig. 7G).

The HE staining results revealed that there were no significant pathological alterations in the organ tissues following BT-EXO-CAP treatment. Additionally, ALT, AST, BUN, CREA, CK, and CK-MB expression levels exhibited no significant differences compared to the controls (Fig. 7H-I). Therefore, BT-EXO-CAP and CAP exhibited no significant toxicity levels.

## Discussion

Osteosarcoma (OS), a type of bone cancer, is the most frequently occurring malignancy of the bone [11]. It predominantly affects individuals in the adolescent or pediatric age group, with a prevalence rate ranking it as the third most common cancer in this demographic [12]. The OS occurrence reaches its highest point during adolescence and around the age of 60 [13]. Upon initial diagnosis, ~ 20% of individuals globally exhibit OS metastasis, with the lungs serving as the predominant site of metastasis, comprising 90% of occurrences [14]. Currently, the principal approach for managing OS involves a multimodal treatment strategy comprising preoperative adjuvant chemotherapy, surgical resection, and postoperative adjuvant chemotherapy [15–17]. This approach has led to a rise in the 10-year survival rate for patients from 30 to 50% [18].

Nevertheless, the survival rates for patients diagnosed with OS have not demonstrated significant improvement and have reached a point of stability [19]. Additionally, ~ 30–40% of patients experience recurrent OS within 1–2 years following surgical intervention [20]. The prognosis for patients who experience recurrence is unfavorable, as evidenced by a survival rate of 23–29% beyond five years following their second diagnosis [21, 22].

Furthermore, the long-term survival rates after recurrence are below 20% [23]. Multidrug adjuvant chemotherapy regimens are employed to enhance the survival rates of patients with OS incorporating doxorubicin, cisplatin, high-dose methotrexate, and isocyclophosphamide as antagonists [24, 25]. Nevertheless, administration of chemotherapy in high doses may result in critical and potentially fatal adverse effects [26]. Consequently, there exists a pressing necessity to create innovative agents that are less toxic and more biocompatible for the purpose of treating OS.

Extracellular vesicles (EVs) are a type of naturally occurring vesicles that are derived from cells and consist of phospholipid bilayers [27]. These EVs can be classified into three primary subtypes, namely microvesicles, EXOs, and apoptotic bodies. EVs are responsible for encapsulating cell-derived contents and mediating intercellular communication locally and over long distances transportation of functional substances, including RNA, DNA, proteins, and lipids [28, 29]. These entities have the potential to serve as biomarkers, therapeutic agents, and drug delivery vehicles [30]. EVs are involved in intercellular communication, signal transduction, and tumor metastasis. Some research studies have been conducted to explore the potential of EVs in the treatment of various disorders, including cancer [31], inflammatory diseases [32], neurological disorders [33], myocardial infarction [34], and stroke [35]. EVs possess significant potential as pharmaceutical agents for disease treatment. To enhance the tumor-targeting ability of EVs, Ellipilli et al. [36] employed engineered EXO-like bio-NPs to treat hepatocellular carcinoma by targeting liver tumor stem cells (CSCs). Ferreira et al. [37] have devised biocompatible tumor cell cytosolic EXO-mimetic porous silica nanoparticles (PSiNPs) to serve as drug carriers to target cancer chemotherapy and found that the PSiNPs were used in somatic Both cancer cells and CSCs exhibited significant cellular uptake and cytotoxicity. Our team will try to construct multifunctional NPs with enhanced OS targeting ability in future studies.

The lung is the most prevalent site of OS metastasis, and the symptoms that follow lung metastasis are an important mortality cause in patients [3]. Interestingly, we found that BT-EXO-CAP has a high aggregation concentration in lung tissue, which may be due to BT-EXO-CAP targeting metastatic OS cells in the lung, and we are studying to follow up on this point.

Ferroptosis is a distinct form of cell death and reduced mitochondrial cristae, ferrous iron overload, and lipid peroxidation are important features of cellular ferroptosis [38]. Morphological changes in OS cells after BT-EXO-CAP treatment are in line with the conventional ferroptosis morphology and can be rescued by Fer-1 [39]. Previous studies have shown that TFRC suppression inhibits ferroptosis, and its overexpression may elevate iron levels, thereby increasing ferroptosis susceptibility [40]. Furthermore, ferritin, consisting of FTL and FTH, plays a crucial role in iron storage and maintenance of iron homeostasis [41]. Our results suggest that BT-EXO-CAP simultaneously upregulates TFRC expression and downregulates FTH expression, aiming at promoting ferroptosis.

The Keap1/Nrf2/GPX4 pathway provides a robust defense mechanism against ferroptosis in cells [42]. The Nrf2 transcription factor contributes to mitigating oxidative stress by orchestrating several cytoprotective gene activations which encode cytoprotective and antioxidant enzymes. Typically, Nrf2 binds to the cytoplasmic negative regulator Keap1 and experiences quick ubiquitination-mediated degradation [43]. Upon encountering oxidative stress, Nrf2 undergoes dissociation from Keap1 and subsequently relocates to the nucleus, where it initiates transcriptional activation of genes that harbor antioxidant response elements (ARE) in their promoter regions, including GPX4 and HO-1 [44]. The GPX4 protein is classified as a selenoprotein and functions as a GSH-dependent peroxidase, aiming at counteracting lipid oxidation in membranes [45]. The reduction of GSH levels has been observed to decrease the activity of GPX4, a crucial event upstream of mitochondrial dysfunction, ultimately resulting in the occurrence of ferroptosis [46]. Consequently, the GPX4 activity decrease is regarded as a ferroptosis indication, and endeavors to reinstate GPX4 expression and activity are a crucial approach to impede ferroptosis. These results suggest that the Nrf2/GPX4 signaling pathway-ferroptosis plays a key role as a “death switch” in the BT-EXO-CAP approach to treat OS. Interestingly, the RNAseq results also suggest that BT-EXO-CAP regulation of the Keap1/Nrf2/GPX4 axis affects OS stem cell proliferation, which is another central question to be answered in our future work.

## Conclusions

**Fig. 8:**
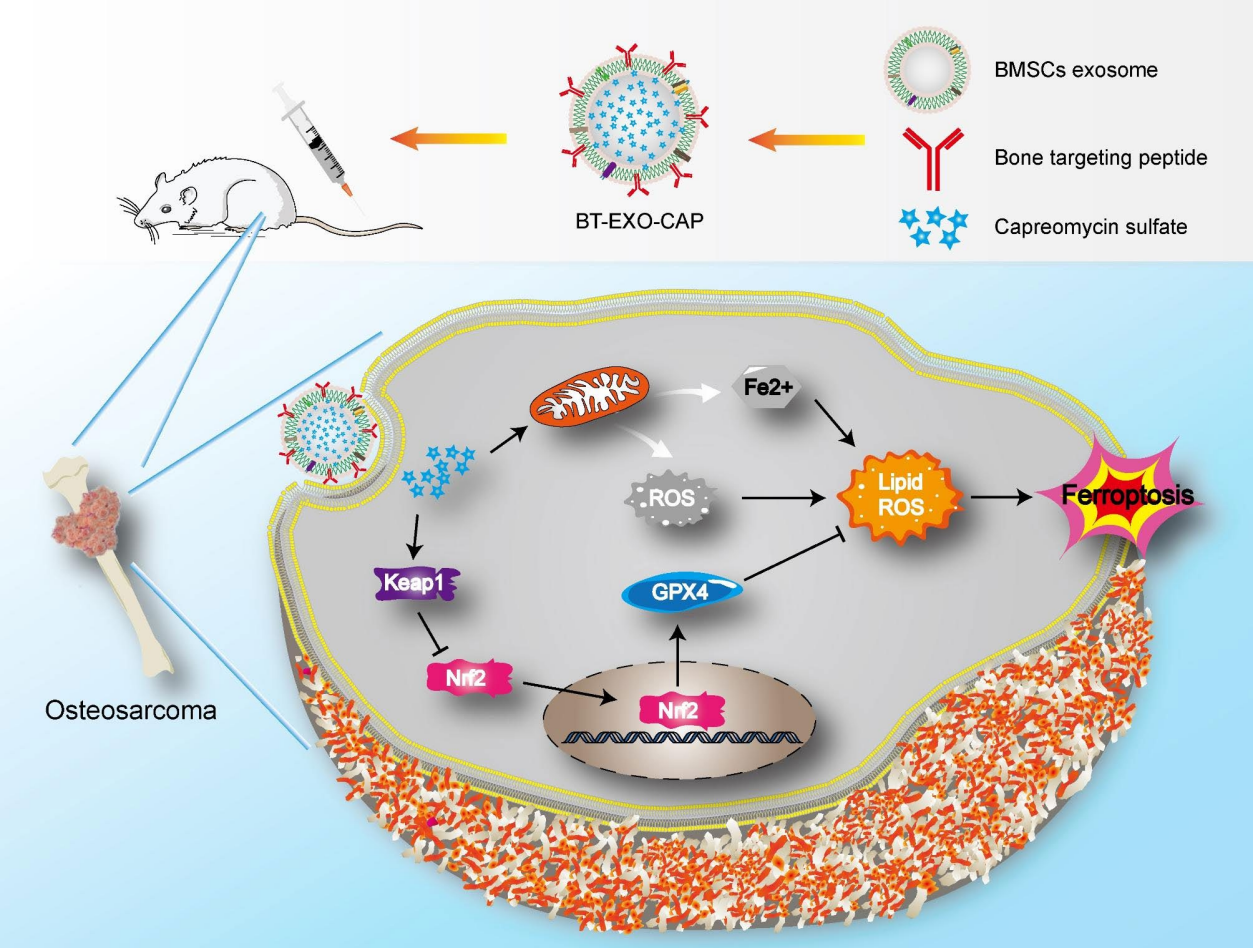
Schematic illustration of BT-EXO-CAP activated Keap1 / Nrf2 / GPX4 signaling pathway leading to ferroptosis in OS cells

In summary, we successfully constructed an emerging NP to obtain OS-targeting ability by modifying BMSCs-derived EXOs with bone-targeting peptides. This NP can achieve in vivo OS presentation, inhibit tumor growth, and improve survival without significant side effects. In addition, our results suggest that activation of the Keap1/Nrf2/GPX4 signaling pathway by this NP induces ferroptosis in OS (Fig. 8). This study provides a new and promising approach for combining ferroptosis-based chemotherapy with targeted therapy for OS.

## Methods

### Cell culture

The human OS cell lines 143B, MG63, Saos-2, and U2-OS (Procell, China) were subjected to culture in a high-glucose Dulbecco’s Modified Eagle Medium (DMEM) (Hyclone, USA) that contained 10% fetal bovine serum (FBS) (HyClone, USA) and 1% penicillin/streptomycin (HyClone, USA) at 37 °C in 5% CO_2_. Human BMSCs (Hm-BMSCs, Procell, China) went through culture in alpha-MEM (Hyclone, USA) containing 10% FBS (HyClone, USA) and 1% penicillin/streptomycin (HyClone, USA) at 37 °C in 5% CO_2_.

### EXO preparation

The purification of EXO was carried out following previously established protocols, albeit with certain changes [47]. The conditioned cell culture medium that contained EXO was subjected to 15 min centrifugation at 3,000 g at 4 °C, aiming at eliminating cells. The resulting supernatant was subjected to 60 min centrifugation at 100,000 g at 4 °C, washing with cold PBS, and another 60 min centrifugation at 100,000 g at 4 °C. EXO was resuspended in PBS and preserved at -80 °C.

The study determined EXO concentration by measuring protein content through Pierce BCA Protein Assay Kit (Thermo, USA). The EXO samples were diluted to a concentration of 1 mg/mL and subjected to size distribution analysis employing the Mastersizer instrument (Malvern, UK). The study employed transmission electron microscopy (TEM) (Hitachi HT-7800, Japan) to observe the morphology. Additionally, Western blotting (WB) was utilized for determining EXO marker expressions, CD9/63/81, and TSG101.

### Modification of EXO with bone-targeting peptide

The Sener Biological Technology (Hefei, China) synthesized bone-targeting-peptide (98% purity assayed by HPLC), which was then dissolved in a solution of 5 mM Tris(2-carboxyethyl) phosphine hydrochloride (TCEP) at 1 mg/mL. Subsequently, Xi’an Rixi Biological Technology Company (Xi’an, China) synthesized 1,2-distearoyl-sn-glycero-3-phosphoethanolamine-N-[methoxy(polyethyleneglycol)-2000]-maleimide (DSPE-PEG-Mal, 98% purity assayed by HPLC), which was then dissolved in a 5 mM HEPES buffer at 1 mg/mL. DSPE-PEG-Mal was subsequently utilized to react with the peptide in a molar ratio of 1:1 at room temperature for 24 h, aiming at preparing DSPE-PEG-Mal-Cys-SDSSD. The mixture went through 24 h dialysis against distilled water using a dialysis bag (molecular weight cutoff of 3000 Da). Eventually, the resultant post-dialysis solution experienced freeze-drying, following which the DSPE-PEG-Mal-Cys-SDSSD powder was preserved at − 20℃ [7].

For the purpose of combining EXO with bone-targeting peptide, a solution consisting of 10 μL of EXO (10^12^ particles/mL) and 90 μL of the DSPE-PEG-Mal-Cys-SDSSD (10 μM) was gently introduced into 100 μL PBS. The resulting mixture was then subjected to an overnight incubation at 4℃. For the removal of uncombined DSPE-PEG-Mal-Cys-SDSSD, the mixture was subjected to PBS washing by 70 min ultracentrifugation at 10^5^ ×g at 4℃ and was then resuspended in PBS [7].

### Drug loading into BT-EXOs

The drug loading process occurred following the previously described methodology [48]. In brief, the combination of BT-EXO and CAP was subjected to sonication using specified settings as the experimental protocol involved the application of 20% amplitude for a total of 6 cycles consisting of 30 s on/off for three minutes with a two-minute cooling period between each cycle. The resulting BT-EXO-CAP solution was subjected to 60 min incubation at 37 °C to enable the exosomal membrane recovery as well as to eliminate any excess-free drug through ultracentrifugation. The study employed UV-Spectroscopy (Omega POLARstar, Germany) to measure the free CAP concentration in the supernatant at the RF λmax (268 nm) [49].

### Drug release assay

The experiment involved immersing dialysis tubes in PBS solutions with pH values of 7.4 and 4.5, followed by the determination of the in vitro drug release profile by transferring 3 mL of BT-EXO-CAP solution into the tubes [50].The release of CAP into the bathing medium was assessed at 37 °C over 48 h at the RF λmax (268 nm) using UV-Spectroscopy (Omega POLARstar, Germany) at time intervals of 1, 2, 4, 6, 12, 18, 24, 36, and 48 h.

### Flow cytometry

The cell culture was maintained in six-well plates and subjected to various treatments for 24 h. Following this, the cells were collected and subjected to 30 min staining with FerroOrange dye (#F374, Dojindo Laboratories) or 10 μM C11-BODIPY™ 581/591 (#D3861, Thermo Fisher Scientific, USA). The cells were exposed to overnight fixation in cold 70% ethanol at − 20 °C, followed by staining with propidium iodide for the purpose of conducting cell cycle analysis. Flow cytometry was conducted (Beckman Cytoflex LX, USA), and data were analyzed by CytExpert software.

### Cytotoxicity assay

The experiment involved seeding cells into 96-well plates (1 × 10^4^ cells/well), followed by treatment as specified. The absorbance of reduced WST-8 at 450 nm was measured at different time intervals for calculating the cell numbers in three replicate wells.

### Colony formation

The measurement of colony formation was conducted following the previously outlined methodology [51]. Six-well plates were utilized for cell seeding (200 cells/well), and the cells were then subjected to 7-day culture and 20 min fixation with ice-cold methanol prior to being stained with crystal violet. Colonies containing > 50 cells were counted utilizing an optical microscope (Carl Zeiss, Germany).

### Wound healing assay

In brief, 5 × 10^5^ cells went through 24 h culture in six-well plates, and a 200 μL sterile pipette was used for scratching the monolayers, followed by taking the photographs after 24 h. The experiment was repeated three times.

### Transwell assay

Matrigel solution was applied to the upper chamber for 2 h (Corning, Australia) while adding a 1 × 10^5^ cells/well cultured in 100 μL FBS-free medium with 600 μL complete medium into the lower chamber. After 24 h, non-migrating cells situated on the upper side of the chamber were eliminated. The invasive cells located in the lower chamber were subjected to 20 min fixation with methanol and subsequent 30 min staining with 0.1% crystal violet (Solarbio, China) prior to being counted through an inverted microscope.

### RNA sequencing

The RNA extraction process involved the use of TRIzol to isolate total RNA from three distinct OS cells. Subsequently, RNA-Seq was conducted on the extracted RNA, and the resulting data were analyzed at the UAB Genomics Core Facility. The RNA-Seq was conducted using the Illumina NextSeq500 platform, following the protocols (Illumina Inc., San Diego, CA).

### Intracellular ROS measurements

The intracellular ROS measurement was conducted through DCFDA/H2DCFDA-Cellular ROS Assay Kit (Abcam, USA). In brief, cells were subjected to 30 min incubation with 20 μM DCFDA at 37 °C. Following this, the cells were washed with PBS, and the resulting fluorescence was detected using an Olympus BX50 fluorescence microscope.

### WB analysis

The experiment involved the separation of equivalent quantities of protein per well through the use of 8–12% sodium dodecyl sulfate-polyacrylamide gel electrophoresis. The separated protein was then transferred to polyvinylidene fluoride membranes (Millipore, USA). The membranes went through incubation with primary antibodies that were specifically raised against the subsequent proteins: β-Actin (1:3000, Cat#ab20272, Abcam), CD63 (1:1000, Cat#ab68418, Abcam), TSG101 (1:1000, Cat#ab125011, Abcam), CD9 (1:1000, Cat#ab236630, Abcam), CD81 (1:1000, Cat#ab79559, Abcam), calnexin (1:1000, Cat#ab22595, Abcam), ACSL4 (1:10000, Cat#ab155282, Abcam), TFRC(1:1000, Cat#ab214039, Abcam), ALOX12(1:1000, Cat#ab168384, Abcam), GPX4(1:1000, Cat#ab125066, Abcam), SLC7A11(1:1000, Cat#ab307601, Abcam), FTH1(1:1000, Cat#ab75972, Abcam), Keap1(1:1000, Cat#ab227828, Abcam), Nrf2(1:1000, Cat#ab62352, Abcam), PCNA(1:5000, Cat#ab92552, Abcam).

### Cellular GSH levels

The GSH measurement was conducted by a kit, per the protocols (Beyotime, China). The cells were cultured in 6-well plates, followed by adding lysate and centrifuging the medium at 10,000 g for 10 min. The GSH concentration in the supernatant was determined by referencing a standard curve.

### Nrf2 overexpression lentivirus transfection

The 143B cells in the logarithmic growth stage went through seeding into 6-well plates. Upon reaching 60% cell density, 2 μL GFP-NC or GFP-Nrf2 lentivirus solution (Haixing Biosciences) was introduced into the cell medium, respectively. After 24 h, the Nrf2 stably overexpressed cells were screened by the addition of puromycin at a concentration of 2 μg/ml.

### Animal experiments

The study utilized male BALB/c nude mice (5-week-old) procured from Xiamen University Laboratory Animal Center (Xiamen, China). The mouse were housed in a temperature-controlled environment (24 ± 1℃) with a 12 h light/12 h dark cycle, and an air filter cover was provided. The under-anesthesia mice received an injection of 143B cells (2 × 10^6^ /10 μL) into the right tibia and were allowed to grow for 7 days, aiming at constructing the nude mouse OS model, while injecting saline with BT-EXO (1 × 10^8^/mL) and CAP (20 mg/kg) through the tail vein. A total of 68 mice were divided into 4 groups for the experiment. The Xiamen University Laboratory Animal Management and Ethics Committee granted ethical approval (No. XMULAC20200148) following the animal welfare guidelines of the Chinese Society of Laboratory Animals.

### Cytotoxicity evaluation

The serum of mice was analyzed for the presence of alanine transaminase (ALT), aspartate transaminase (AST), blood urea nitrogen (BUN), creatinine (CREA), creatine kinase (CK), and creatine kinase-MB (CK-MB). Following euthanasia, the organs, including the heart, liver, spleen, lung, and kidney, were extracted for H&E staining.

### Statistical analysis

The data have been presented in the form of mean ± SEM. The statistical analysis employed in this study involved the utilization of the Student’s t-test and one-way ANOVA for comparing two-group and among more than two groups, respectively. p < 0.05 indicated statistically significant differences. * p < 0.05, ** p < 0.01, *** p < 0.001.

### Electronic supplementary material

Below is the link to the electronic supplementary material.

Supplementary Material 1

## Data Availability

The data that support the findings of this study are available from the corresponding authors upon reasonable.

## Abbreviations

ALT: Glutamic-pyruvic transaminase
AST: Glutamic oxalacetic transaminase
BMSCs: Bone marrow mesenchymal stem cells
BT-EXO: Bone-targeting-exosomes
BUN: Blood urea nitrogen
CAP: Capreomycin
CCK-8: Cell counting kit-8
CK-MB: Creatine kinase-MB
CK: Creatine kinase
CREA: Creatinine
DMEM: Dulbecco’s Modifed Eagle Medium
EXOs: Exosomes
FBS: Fetal bovine serum
GPX4: Glutathione peroxidase 4
NP: Nanoparticle
OS: Osteosarcoma
ROS: Reactive oxygen species
TEM: Transmission electron microscopy
