# Supplementary material for: Bone-targeting exosome nanoparticles activate Keap1 / Nrf2 / GPX4 signaling pathway to induce ferroptosis in osteosarcoma cells
Source: J Nanobiotechnology. 2023 Sep 30;21:355. doi: 10.1186/s12951-023-02129-1 (PMC10541697; doi:10.1186/s12951-023-02129-1)
Supplement: Supplementary file 1 — Supplementary Material 1 [file 12951_2023_2129_MOESM1_ESM.pdf]

Supplementary figures and figure legends

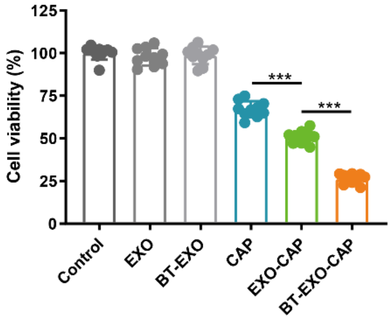

Figure S1. The effect of Exosomes, Bone targeting exosomes and Capreomycin on the activity of OS cells. *** *P* <0.001.

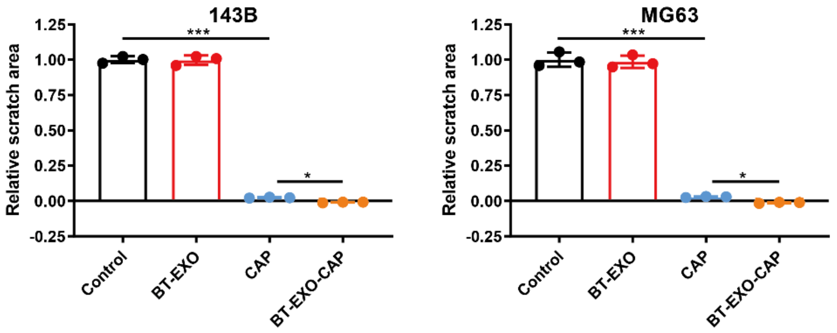

Figure S2. Statistics of cell scratch experiments. * *P* <0.05, *** *P* <0.001.

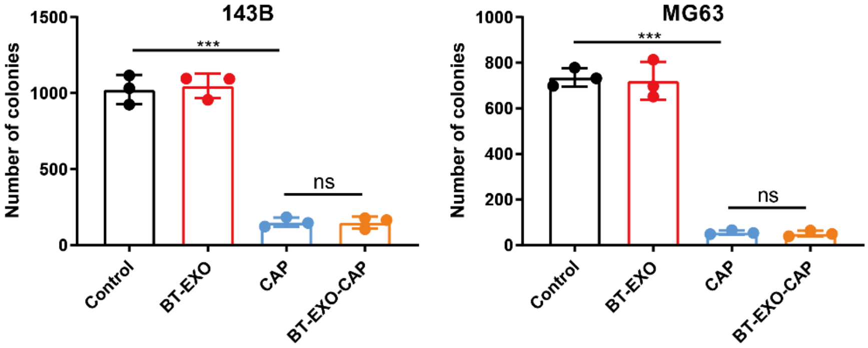

Figure S3. Statistics of cell cloning experiments. *** *P* <0.001.

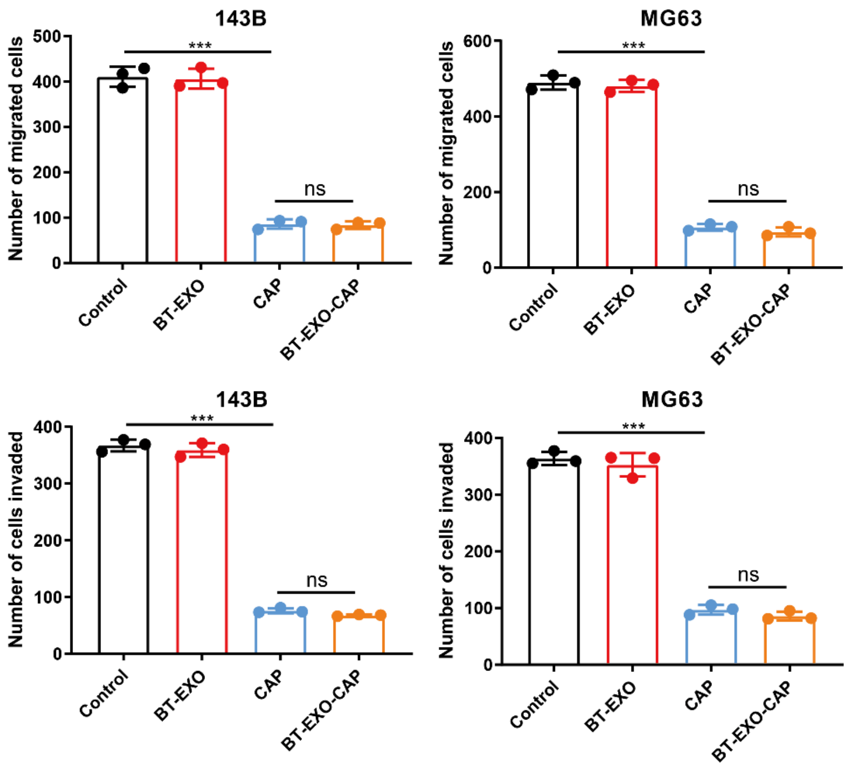

Figure S4. Statistics of cell migration and invasion experiments. *** *P* <0.001.

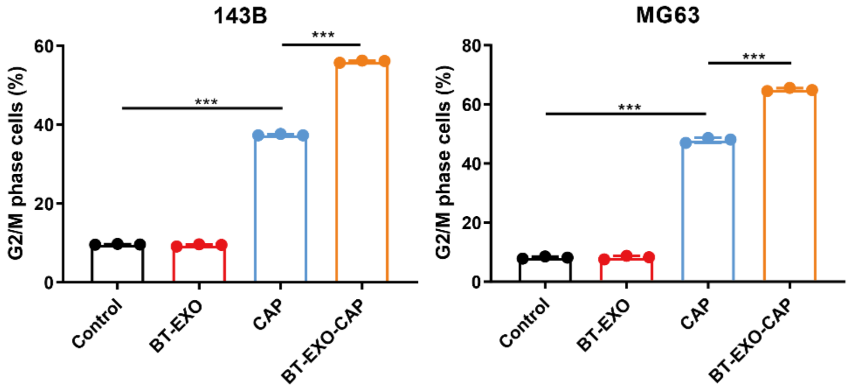

Figure S5. Statistics of the cell cycle at G2/M phase. *** *P* <0.001.

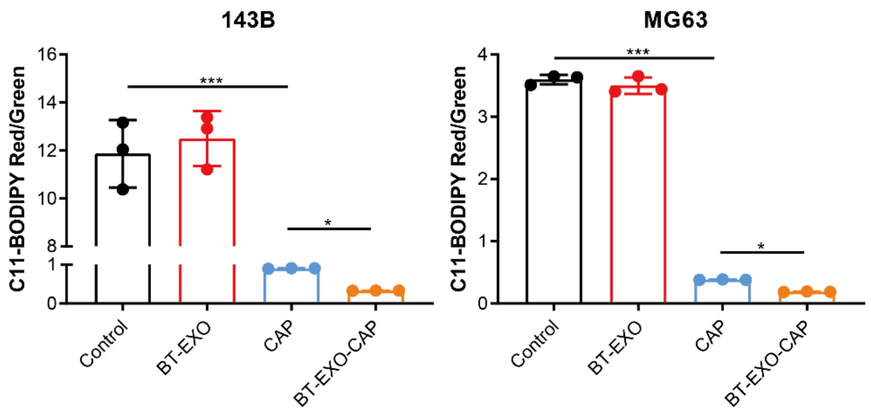

Figure S6. The proportion of C11-BODIPY red cells to green cells. * *P* <0.05, *** *P* <0.001.

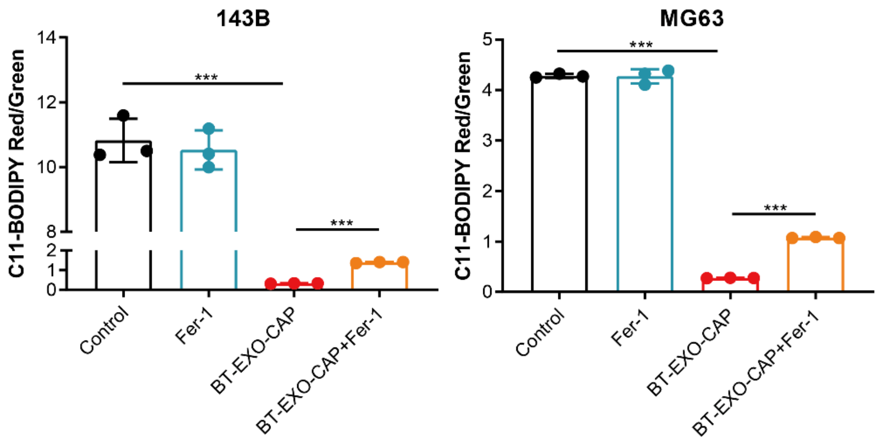

Figure S7. The proportion of C11-BODIPY red cells to green cells. *** *P* <0.001.

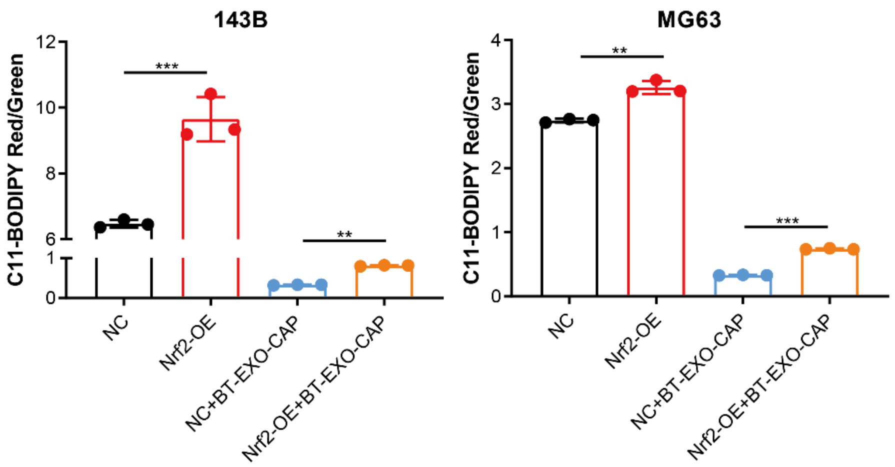

Figure S8. The proportion of C11-BODIPY red cells to green cells. ** *P* <0.01,*** *P* <0.001.
